# Evapotranspiration depletes groundwater under warming over the contiguous United States

**DOI:** 10.1038/s41467-020-14688-0

**Published:** 2020-02-13

**Authors:** Laura E. Condon, Adam L. Atchley, Reed M. Maxwell

**Affiliations:** 1 Department of Hydrology and Atmospheric Sciences, University of Arizona, Tucson, AZ USA; 2 Earth and Environmental Sciences, Los Alamos National Lab, Los Alamos, New Mexico, USA; 3 Integrated Groundwater Modeling Center, Department of Geology and Geologic Engineering, Colorado School of Mines, Golden, CO USA

**Keywords:** Climate change, Hydrology, Hydrology

## Abstract

A warmer climate increases evaporative demand. However, response to warming depends on water availability. Existing earth system models represent soil moisture but simplify groundwater connections, a primary control on soil moisture. Here we apply an integrated surface-groundwater hydrologic model to evaluate the sensitivity of shallow groundwater to warming across the majority of the US. We show that as warming shifts the balance between water supply and demand, shallow groundwater storage can buffer plant water stress; but only where shallow groundwater connections are present, and not indefinitely. As warming persists, storage can be depleted and connections lost. Similarly, in the arid western US warming does not result in significant groundwater changes because this area is already largely water limited. The direct response of shallow groundwater storage to warming demonstrates the strong and early effect that low to moderate warming may have on groundwater storage and evapotranspiration.

## Abstract (web summary)

New hydrological simulations show for the first time how sensitive groundwater and surface water connections are to systematic warming across the continental United States. The authors here show a clear reduction in subsurface water storage under a warming climate and intensified aridification of north America.

## Introduction

Warming temperatures are systematically shifting the balance between water and energy drivers of terrestrial systems^1–3^. In the US, the climatologically defined 100th meridian, where water demand is roughly balanced by supply, has shifted eastward since the 1980’s; essentially increasing the area of the Western US where evapotranspiration is limited by water availability^1^. Also, over the 20th century forest growth has become more limited by water availability than energy, forests have experienced greater vapor deficit stress^2^, and the relationship between temperature and vegetation productivity has been weakening^3^. Trends which are consistent with greater water limitation.

Despite these findings, the extent to which temperature and water availability will control ecosystem function in the future remains uncertain^4,5^. A recent study noted an increase in groundwater droughts coincident with hot periods in the 21st century (as opposed to only dry periods) and inferred that this shift was likely do to evaporative shifts with warming^6^. Higher temperatures increase evaporative demand, but how much this will induce moisture deficits and evaporative stress, as opposed to increased evapotranspiration, is partially dependent on climate^7–9^. Plant water availability is controlled by local precipitation and soil moisture, but soil moisture and streamflow may also be supported by shallow groundwater^10,11^.

Thirty percent of the total renewable freshwater supply gets recharged to groundwater annually^12,13^. Groundwater depth and lateral redistribution of water in the subsurface can control the partitioning of evapotranspiration runoff and recharge^14,15^, as well as the sensitivity of the land surface to changes in precipitation and temperature^9^. Groundwater is often the slowest moving part of the terrestrial water cycle; it acts as a reservoir providing water for plants during rainless periods contributing to decadal oscillations in total terrestrial water availability^10,16^.

In a warming climate, increased evapotranspiration may shift the fraction of precipitation that runs off as surface water or infiltrates to the subsurface as recharge. Long-term shifts in recharge patterns can change groundwater levels and subsequently groundwater surface water interactions and soil moisture^17,18^. Despite these established interactions, quantifying the role of groundwater in a warming climate remains a significant challenge^19,20^. Existing large-scale models often exclude or oversimplifying groundwater storage dynamics and their contributions to surface water availability^5,16^. A comparison of global hydrologic models with GRACE satellite estimates of groundwater storage trends found that models significantly underestimate decadal trends in water storage due to a lack of groundwater representation^16^. Consistent with these findings, many have stressed the need to incorporate groundwater processes into large-scale hydrologic simulations in order to improve prediction^10,21,22^.

Here we apply an integrated, high-resolution surface-groundwater model over most of continental North America to conduct a controlled numerical experiment designed to isolate the impact of increased temperature due to climate change on evapotranspiration and groundwater storage. This is the first evaluation of climate change impacts using a hydrologic model that explicitly simulates physically based lateral groundwater flow and groundwater surface water interactions across the US. By explicitly simulating groundwater dynamics in a series of pseudo-warming experiments we seek to directly evaluate both the impact of warming on subsurface storage, and the role of groundwater in system response to warming for the first time.

## Results

### Warming increases evaporative demand

We compare a historical baseline climate scenario with three perturbed scenarios with uniform warming of 1.5, 2, and 4 °C. The purpose of the uniform so-called, pseudo-warming^23^ perturbations applied here is to directly evaluate the sensitivity of the terrestrial hydrologic system to varying degrees of warming in a series of controlled experiments. By systematically isolating the integrated hydrologic response to warming, our modeling approach can quantify the importance of dynamic groundwater interactions at large scales. We acknowledge that climate projections show spatially variable warming trends, and temporal variability in warming especially with extreme events^24^. Our goal is not to capture this variability but to evaluate response to a range of long-term warming possibilities. Incorporating transient and heterogenous projections would limit our ability to isolate the critical hydrologic response to warming.

The three warming scenarios selected here span the range of projections for the CONUS over the 21st century; 1.5 °C is the expected warming by the middle of the 21st century if current warming trends continue^25^, 2 and 4 °C further provide a range of possible warming scenarios by the end of the 21st century^24^. Some parts of the country are projected to warm faster or slower than these national averages. For example, the Northeast is projected to warm faster than the Southwest^24,26^. Therefore, our 4 °C scenario may be a shorter-term likelihood for some parts of the country than others. The three temperatures chosen here are not intended to be national projections for a specific time period. We seek to quantify the response to warming separate from considerations of variability and uncertainty in warming projections therefore these values were chosen to represent a range of warming that is reasonable across CONUS.

In all scenarios, the same historical observed precipitation is applied in order to isolate impacts of temperature increases from shifts in water supply, which may occur with changes in precipitation. We focus on warming trends for this study because they are generally more certain than precipitation projections, which can be highly variable^27,28^. All tests start from the same initial groundwater configuration developed based on modern climate (i.e. 1950–1999)^14,29^. Each simulation is run for 4 years repeating a single year of historical observed meteorological forcings four times to control for interannual variability (refer to Methods section for additional details).

Aridity measures the relative balance between water demand (calculated here as potential evapotranspiration, PET) and water supply (precipitation, P). The Aridity Index (AI, PET/P) can be used to characterize the water and energy drivers of hydrologic systems. Where the aridity index is <1, precipitation is greater than evaporative demand and the system is energy (as opposed to water) limited. In the US the 100th meridian corresponds with an AI of one and roughly divides the country into the more arid west and the more humid east^1^. Moving from west to east across this gradient there are stark differences in both natural ecosystem composition, agricultural practices, and crop choice^1^.

**Fig. 1:**
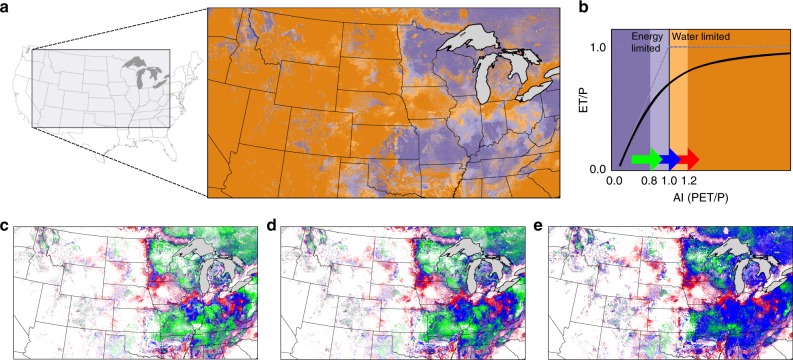
Warming increases aridity and shifts water and energy limits of Evapotranspiration. **a** Map of Baseline aridity index (AI) classified as strongly water limited (dark orange), weakly water limited (light orange), weakly energy limited (light purple), and strongly energy limited (dark purple). Shifts between AI classifications for the 1.5 °C (**c**) 2 °C (**d**) and 4 °C cases (**e**) (color legend shown with the arrows on **b**). The Budyko relationship between AI and evaporative fraction (**b**) (solid black line), plotted relative to the water (Evapotranspiration (ET)/Precipitation (P) = 1) and energy (Evapotranspiration (ET) = Potential Evapotranspiration (PET)) absolute limits (dashed blue lines).

Aridity is also a predictor of watershed partitioning; the Budyko hypothesis relates the relative fraction of precipitation that leaves a watershed as evapotranspiration (as opposed to runoff) to aridity^7,30^. Using long-term observations, Budyko showed that a single curvelinear relationship, the Bukdyo Curve between aridity index and evaporative fraction (i.e. the portion of incoming precipitation that leaves a watershed as Evapotranspiration (ET) rather than streamflow) could capture 90% of the variability between the major watersheds studied^7^. An aridity map of the baseline simulation clearly highlights the transition from water to energy limited systems moving from west to east (Fig. 1a).

Potential evapotranspiration, defined as the amount of evaporation which would occur absent any water limitation, is controlled by incoming radiation, temperature gradients, relative humidity, and wind. As we progressively warm the system PET increases across the domain (Fig. 1b–d). Despite a spatially uniform warming perturbation, the PET changes are spatially variable due to non-linear relationships between temperature, specific humidity and PET. Because precipitation is held constant, increases in PET translate directly to increased aridity in the domain. The difference maps in Fig. 1 show locations where increases in aridity shifts the degree of water or energy limitation. In the arid western US, increased PET only exacerbates an already water limited system. East to the 100th meridian, however, warming can change the balance between water supply and demand. Most notably, in an expanding band roughly along the 100th meridian we show shifts from weekly water limited systems to strongly water limited systems and transitions from energy to water limited systems. This finding is consistent with a recent study that showed an eastward movement of the symbolic 100th meridian as warming increases aridity across the great plains of the US^1^.

**Fig. 2:**
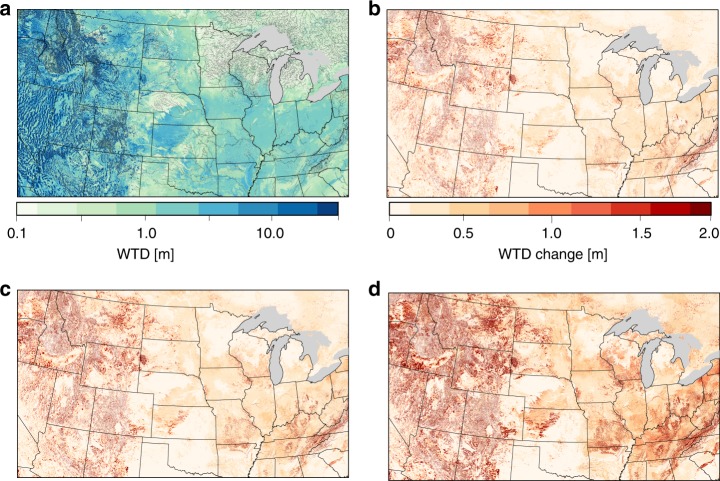
Warming increases water table depth. **a** Map of the monthly average Water Table Depth (WTD) for the last month of the Baseline simulation compared to the difference the final WTD (warming – Baseline) for each of the warming scenarios; 1.5 °C (**b**), 2 °C (**c**), and 4 °C (**d**).

Increased aridity results in a drying trend in the subsurface. Water table depth, is the distance from the land surface to saturated groundwater. The baseline groundwater configuration (Fig. 2a) was generated using the average climate conditions for the second half of the 20th century (refer to Methods for additional details). The baseline water table depths represent a groundwater configuration that is in equilibrium with modern climate conditions; generally speaking, in the more humid portions of the US the water table is shallower and more closely follow topography whereas in the more arid west groundwater is generally deeper^31,32^. With warming induced increases in aridity, groundwater depths increase across the domain demonstrating a systematic drying of the subsurface (Fig. 2b–d). Here we evaluate the connections between evaporative demand, groundwater storage, and evaporative response to stress that results in these changes.

### Evaporative response varies with aridity

**Fig. 3:**
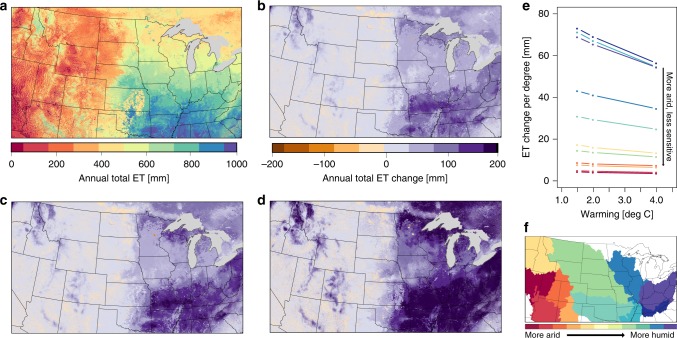
Evapotranspiration is most sensitive to warming in the eastern US. **a** Map of Baseline annual evapotranspiration (ET) and the annual change in ET (warming – Baseline) for each of the warming scenarios; 1.5 °C (**b**), 2 °C (**c**), and 4 °C (**d**). All maps are for the final year of simulation. Aggregated ET changes normalize by the degree of warming (Change in ET/degrees of warming) (**e**) are summarized by the major US watersheds in the domain colored by their rank with respect to average baseline aridity (**f**).

The extent to which increased evaporative demand induces actual increases in ET, as opposed to simply increasing evaporative stress, is largely a function of the water available to meet increased demand. For our tests, ET increases most in the eastern US where the baseline aridity would indicate an energy limited system (Fig. 3a–d). In the more arid western portions of the domain there is already a strong water limitation therefore, increased demand does not impact the actual ET flux in the system. In some parts of the west, there are actually small declines in ET with warming as the water limited portion of the year expands and the system dries further limiting water availability in these already dry locations. The areas of the western US with the largest increase in ET correspond to mountainous areas where ET increases are caused by changes in snowpack and melt timing. While the land surface model used here does simulate snowpack and snowmelt, the impacts of warming on high elevation snow-driven systems are a topic of much research and outside the scope of the present discussion (e.g. refs. ^33–35^).

As the degree of warming increases from 1.5 to 4 °C across our simulations, ET changes remain small in the western portions of the domain but are exacerbated in the Eastern US (Fig. 3b–d). This larger overall ET response with greater warming is especially clear in the southeast, where water availability is largest.

While the total ET impact increases with additional warming, the sensitivity to incremental warming (i.e. the change in ET per degree celsius of warming) decreases. Aggregating across the major watersheds covered in the domain (Fig. 3f), we find that ET response per degree of warming is always lowest in the 4 °C scenario and highest in the 1.5 °C case (Fig. 3e). This highlights the physical limitations on the system; increased warming can only sustain higher ET when the water supply can also keep up. This point is also illustrated by the slope of the ET response lines. The eastern basins, where ET increase are largest, also have the greatest change in response from the 4 °C to the 1.5 °C cases, showing increased water limitation as the severity of the warming increases. In the western basins where ET response to warming is low, the response per degree of warming stays relatively constant as the degree of warming increases.

### Warming decreases groundwater storage

**Fig. 4:**
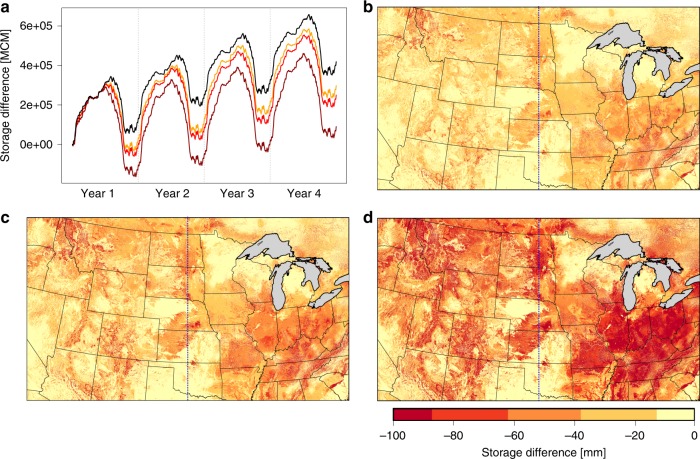
Warming results in groundwater storage losses. **a** Timeseries of the daily total storage relative to the initial storage (baseline in black, 1.5° in orange, 2 °C in red, and 4 °C in dark red). Maps of the ending storage difference between each of the warming scenarios; 1.5 °C (**b**), 2 °C (**c**), and 4 °C (**d**) relative to the Baseline ending storage. The dashed blue line is the center of the domain dividing east and west.

Precipitation is intentionally held constant between years and across all simulations to isolate the impact of increased evaporative demand across scenarios. Thus, within this framework, increases in ET must be supported by shifts in the partitioning of incoming precipitation between streamflow and ET and changes in storage. The baseline simulation starts from a state of dynamic equilibrium (<3% change in storage relative to incoming precipitation across the domain^14^). Over the 4-year simulation period there is a strong seasonal signal in the baseline run, as groundwater is recharged in the winter and discharged in the summer. There is also a small positive trend in the baseline case indicating that the baseline system has a net storage gain from year to year (Fig. 4a). All simulations start from the same initial storage but diverge over time. The warming simulations are all characterized by decreased storage (indicated by the downward shift of the warming scenario lines relative to baseline). By the end of the 4th-year the three warming runs have lost 119,000 Million Cubic Meters (MCM), 167,000 MCM, and 324,000 MCM for the 1.5, 2, and 4 °C cases respectively. For reference, these groundwater losses are between 4 and 10 times the volume of Lake Powell (30,000 MCM) and between 25% and 68% the volume of Lake Erie (480,000 MCM). Also, seasonal storage cycles are amplified with warming. The annual recharge (i.e. the storage difference from the first day of each year to the day of peak storage) increases by 16%, 24%, and 49% for the 1.5, 2, and 4 °C cases respectively comparing between the first and last years of each scenario. These changes show that even the most moderate warming projection (1.5°) can shift groundwater surface water exchanges and lead to substantial and persistent storage losses. Furthermore, consistent with the ET trends, this shows the largest changes in storage per degree of warming for the 1.5 °C case, again highlighting the initial sensitivity of the system to warming and decreases sensitivity as the system dries out.

Storage losses, similar to land surface impacts, are spatially heterogeneous (Fig. 4b–d). The largest groundwater declines occur in the Eastern US, consistent with the areas with the largest increase in ET. However, the contrast in storage response between east and west is not as sharp as with ET changes (Fig. 3b–d). Of the total storage losses, 55–59% occur in the eastern half of the study domain (blue dashed lines, Fig. 4b–d), compared to 85–86% of ET increases occurring in the eastern half. In the east, storage losses are caused by increased ET depleting shallow storage. In the west, the groundwater is already deeper and more disconnected from the land surface; here the storage changes reflect, decreased recharge to the groundwater for the warming cases relative to the baseline.

In addition to regional trends, the smaller scale spatial heterogeneity in the storage response to warming highlights the primary drivers of groundwater configuration: topography, geology, and climate. Changes are smaller in the low-lying areas along streams where overland flow and lateral convergence of groundwater in the subsurface maintains constant storage. Additionally, the spatial patterns illustrate spatially variable soil and geologic units where differences in hydraulic conductivity and texture control the ease with which water moves through the subsurface and can be released from storage.

### Groundwater declines support ET response

**Fig. 5:**
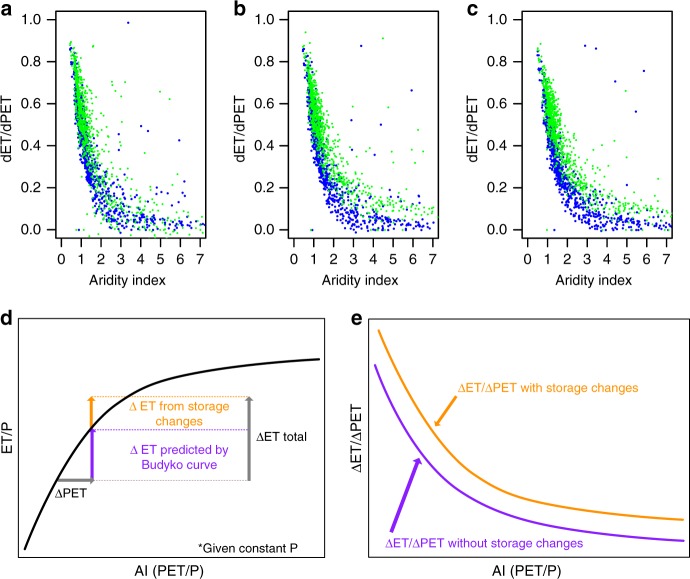
Evapotranspiration sensitivity decreases as system warms and storage changes decrease over time. Scatter plots of the sensitivity to warming (change in evapotranspiration (ET) per change in potential evapotranspiration (PET)) versus aridity index for the first (green dots) and fourth (blue dots) years of simulation for the 1.5 °C (**a**), 2 °C (**b**), and 4 °C (**c**) scenarios. The systematic decrease in sensitivity (i.e. downward shift) can be explained by storage changes increasing water availability relative to the Budyko framework for equilibrium systems (**d**). This storage contribution increases the system response for a given PET perturbation (**e**).

Small shifts in aridity translate to larger changes in ET in energy limited systems than water limited locations. This is illustrated by the large ET response to warming shown for the eastern US and the smaller response in the western US (Fig. 2). Comparing behavior across 1500 watersheds in the domain (defined by USGS HUC8s), we find an inverse and non-linear relationship between ET sensitivity to warming (defined here as change in ET per change in PET) and aridity for all simulations (Fig. 5). This relationship is what would be expected from the slope of the Budyko Curve (Fig. 1c).

However, warming sensitivity is not constant throughout the four-year simulations. We find systematic decreases in ET response to increased demand for the same aridity level between years 1 and 4 of the simulations (illustrated by the downward shift between the blue and green points in Fig. 5. Similar, but less pronounced shifting, can also be seen between intermediate simulation years, we present only the first and last years here for clarity). This shifting indicates that the relationship between ET and PET is shifting over the course of the simulation. In other words, we are not just changing aridity (i.e. moving along the x-axis of the Budyko curve) rather we are also shifting the curve itself as the system dries out.

The Budyko framework assumes that systems are in dynamic equilibrium (i.e. no changes in storage). Thus, shifts in the ET/P ratio that would be predicted by moving along Budyko curve for a given change in aridity index can be interpreted as a prediction of how a system that has re-equilibrated to a different climate might be expected to behave. However, here we consider transient simulations of systems where warming induces changes in storage and there is no expectation that the system is in equilibrium. The systematic decreases in storage are essentially a supplemental water supply in addition to the incoming precipitation, which is held constant for all scenarios and years.

Storage changes are largest in the first year of the simulation and decrease in later years (Fig. 5a). As a result of this additional water supply provided by storage changes, ET sensitivity to changes in PET is systematically larger in the first year of the simulation than the last. This indicates that storage changes decrease the evaporative stress in the system (i.e. the difference between ET and PET). However, the storage changes slow over time as the system dries and re-equilibrates to the warmer temperature state. When this occurs, ET increases more slowly and therefore evaporative stress increases as the system shifts to a more arid state. This is consistent with recent studies that have observed decreased sensitivity to temperature and increased water limitation globally^2,3^.

## Discussion

We show that sustained temperature increases can reduce subsurface storage. Even in the moderate 1.5 °C warming case, subsurface storage decreases by more than 100,000 MCM, a volume larger than most surface reservoirs over the 4-year simulation. We also illustrate that changes in subsurface storage can provide an additional support for ET as the demand (i.e. PET) is systematically increased. This additional supply serves to decrease water limitation on the system and support a larger land surface response to warming especially where groundwater is shallow; 85% of the ET response to warming occurs in the more humid eastern half of the domain. In the arid western portions of the domain, the groundwater is deeper and more disconnected from the land surface. Here increased warming primarily serves to reduce recharge. Potential recharge (calculated as precipitation minus ET) in the western more arid basins decreases by 12–15% relative to the Baseline scenario. This study focuses on storage connections to ET dynamics so the changes to recharge are not discussed in detail here. However, it should be noted that groundwater and baseflow are important to the water supply systems across the western US. Although, the relative increases in ET and declines in recharge are smaller in the west than the east; the heavily managed and often overallocated systems in the west are more sensitive to small changes in water supply.

As the system warms, the eastern US becomes more arid and with this it becomes less sensitive to continued or increased warming. Thus, the response to the 1.5 °C warming case is proportionally larger (i.e. change in storage or ET per degree of warming) than the 4 °C warming case. In the arid west, ET response to warming is low and relatively consistent between cases. Initially the behavior of the humid east is exactly the opposite. However, as the more humid systems warm and dry, they shift to higher aridity values where the sensitivity to incremental warming decreases making the humid east act more like the arid west, given persistent temperature increases. Changes in storage, can help support ET increases for a time, but we show that over the course of our 4-year simulations storage changes slow and so does the ET response to incremental warming.

The non-linear interactions outlined here are not currently represented in other large-scale models. Our approach to isolate the role of groundwater storage given a systematic warming perturbation provides a unique quantification of how groundwater dynamics should be considered in Earth system models. The sensitivity of hydrologic response to shallow groundwater configuration demonstrated here highlights the importance of groundwater as a mediator of system response to change, and the need to understand and incorporate subsurface storage dynamics into long-term watershed projections, especially as climate change is expected to bring about increased extremes, (i.e. not just extreme temperature, but also precipitation). Understanding the role of groundwater as an underlying system mediator to persistent temperature increases is key to understanding how the overall system will respond to these extreme events. Dynamic groundwater interactions will likely affect ecosystem productivity^36^ or conversely be affected by warming induced changes to hydraulic conductivity^37,38^. Also, CO_2_ enrichment may increase water use efficiency and partly compensate for increased evaporative demand caused by warming^28,39^. These interactions were not considered in this study and may counteract some of the sensitivity found here. Never-the-less, the integrated modelling approach used here demonstrates that it is technically feasible to simulate variably saturated lateral groundwater flow at high resolution across large domains; however, this remains a very computationally intensive modeling approach and global models and multi-decadal ensemble simulations are not yet feasible. While currently only one, integrated hydrologic model exists for North America, as more simulations of this type are undertaken a multi-model approach^40^ could be used to further study the impact of conceptual model uncertainty on response or to propagate warming projections to groundwater. Future work should focus on decreasing the computational demands of fully integrated models and using existing large-scale integrated models to validate the groundwater surface water interactions of global earth systems models as new groundwater formulations are implemented in these tools.

## Methods

### Numerical model

All simulations were completed with the integrated hydrologic model ParFlow-CLM. ParFlow-CLM solves 3D variably saturated flow in the subsurface, integrated with physical based overland flow based on the kinematic wave approximation and Mannings equation. Land surface processes are coupled with ParFlow through CLM. The combined model solves the coupled water energy balance at the land surface including; snow accumulation and melt, infiltration, root water uptake, plant transpiration, interception, and bare soil evaporation. Details on ParFlow and ParFlow CLM can be found in refs. ^41–44^. The key difference between this modeling approach and other large-scale simulation platforms (e.g. General Circulation Models and Land surface models) is the explicit calculation of lateral groundwater flow and dynamic interactions (e.g. infiltration and groundwater discharge) between groundwater and surface water at high spatial resolution.

### Domain

The CONUS domain covers roughly 6.2 Million km^2^ in the contiguous US. Grid resolution is 1 km^2^ laterally and the domain extends to 102 m below the land surface using a terrain following grid and variable layer thickness ranging from 0.1 to 100 m. Details on the development of the domain are documented by Maxwell et al.^29^ who developed a steady state groundwater configuration for the domain. Following this work, transient simulations using historical observed meteorology were completed by Maxwell and Condon^14^. This work used, historical observed meteorology for water year 1985 (1 October 1984– 30 September 1985) to drive the ParFlow-CLM model to quasi dynamic equilibrium. Water year 1985 was selected to drive the model, as a representative average year for the domain, without extreme drought or flood conditions. Validation of the baseline simulations of streamflow, groundwater depth and evapotranspiration are provided in the SI of Maxwell and Condon^14^. The CONUS model is designed to simulate modern climate applied to natural hydrologic systems (i.e. without agricultural and urban operations).

### Simulations

The analysis presented here builds from the work of Maxwell and Condon^14^. The Baseline scenario is an extension of the transient simulations from Maxwell and Condon^14^; using the outputs from their analysis as the initial conditions and continuing simulation with the same meteorological forcings. The three warming scenarios start from the same initial condition as the Baseline case and apply the same forcings, except for temperature, which was adjusted uniformly across the domain and all time periods. All four scenarios (Baseline and three warming cases) were run for 4 years. The Baseline simulation is completed in parallel with the warming runs so that any trends in storage due simulation time can be separated from the impacts of the warming perturbation. All simulations are completed at an hourly timestep. For the analysis presented here hourly outputs were aggregated to daily and annual summaries.

The 4-year simulation period was selected to balance the desire for multiyear simulations, with the significant computational expense of large integrated simulations. ParFlow is deigned to run efficiently in parallel using high performance computing resources; however, solving variably saturated flow over large complex domains remains a computationally intensive task. The analysis presented here requires roughly 400,000 core hours per year of simulation running on over 2000 compute cores in parallel.

## Supplementary information

Peer Review File

## Data Availability

The simulations generated and analyzed for the current study will be available in the CYVERSE repository upon publication. Additionally, the simulation platform, ParFlow-CLM is an open source model available on github (https://github.com/parflow).
