# Supplementary material for: Evapotranspiration depletes groundwater under warming over the contiguous United States
Source: Nat Commun. 2020 Feb 13;11:873. doi: 10.1038/s41467-020-14688-0 (PMC7018770; doi:10.1038/s41467-020-14688-0)
Supplement: Supplementary file 1 — Peer Review File [file 41467_2020_14688_MOESM1_ESM.pdf]

Editorial Note: Parts of this Peer Review File have been redacted as indicated to remove third-party material where no permission to publish could be obtained.

Reviewers' comments:

Reviewer #1 (Remarks to the Author):

Review of "Evapotranspiration depletes groundwater under warming" by Condon et al.
Recommendation: Reject, encourage to resubmit
Overall comments:

This article addresses an important topic, namely the effects of regional warming on evapotranspiration and potentially resulting groundwater depletion. However, there is a fundamental flaw in the article:

To simulate warming, the study authors use an offline hydrological model, which they drive with what they call a "uniform pseudo-warming scenario". What they do is to use observations and add respectively, 1.5°, 2° or 4°C on the observed temperatures that are used to drive the model. Unfortunately, these scenarios are not realistic for several reasons:

- Regional warming can be much larger than the global warming. This is clear from several assessments. In many regions, warming may be up to 2-3 times larger than in global mean. The authors cite the IPCC special report on 1.5°C warming, but as can be seen in figures of the report, the mean warming in the United States when global warming reaches 1.5°C is substantially larger, and even more so for extremes (See chapter 3 of the report, Figs. 3.3 and 3.4: https://www.ipcc.ch/site/assets/uploads/sites/2/2019/05/SR15_Chapter3_Low_Res.pdf).

- The authors seem to misunderstand a "1.5°C warmer climate" as a climate in which regional temperature is warming uniformly by 1.5°C. As highlighted above, this is a fundamental misunderstanding. This assumption is reiterated in the text of the article when referring to the IPCC SR15 report: "1.5 deg. C is the expected warming by the middle of the 21st century if current warming trends continue. 2 and 4 deg. C are the projected warming by the end of the 21st century given low and high emissions scenarios respectively". Obviously, these sentences are only true for the global mean warming, not for the regional warming. Overall, land warming is always higher than the global mean warming, because land warms more than the oceans.

- In addition, a uniform temperature anomaly also ignores further geographical or temporal variations in warming. In the highlighted figures from the IPCC SR15 report, it can be seen that some regions of the US warm more than others. In addition, the projected changes in temperature are also varying depending on the season.

For these reasons, I can unfortunately no recommend publication of this article in Nature Communications. However, the topic is certainly interesting. If the authors could drive their simulations with realistic changes in temperature at 1.5°C, 2°C and 4°C global warming, I would recommend to reconsider publication of this article in Nature Communications.

Reviewer #3 (Remarks to the Author):

General comments

The authors have used ParFlow-CLM, a model that has previously been documented and calibrated across the study domain (N America), to 'evaluate the impact of increased temperature due to climate change on evapotranspiration and groundwater depth'. The authors explain that the ParFlow-CLM model has been used as it explicitly represents lateral groundwater flow and interactions between groundwater and surface water at high spatial resolutions in a manner that is not usually captured by GCMs and LCMs. The model simulations were computationally intense which clearly placed limits on the scenarios that could be considered. Nonetheless, the model experiments adequately address the authors' research question.

Condon et al. compare the effect of three uniform warming (or 'pseudo- warming') scenarios applied to a baseline temperature, on groundwater storage, where the warming scenarios span a range of climate change projections for N America. Each simulation uses a single year of historical meteorological forcing repeated for a period of four years: as the authors say 'to control for interannual variability'. It is shown that, in the absence of other change factors, warming reduces groundwater storage, but in a non-trivial manner. Water limited systems become more arid, but in doing so become less sensitive to continued or increased warming. While in less arid areas warming drives groundwater storage losses to support increased evapotranspiration where shallow groundwater is available. However, sustained warming in this context leads to reduced groundwater storage and 'decreasing response to additional warming'.

I enjoyed reading the paper - it is novel and should be of broad interest. It's well written, effectively articulates why the work has been undertaken and the importance of understanding the effect of warming on groundwater storage. The modelling methodology and underlying assumptions are sound, and the results clearly described. The overarching conclusion, that 'nonlinear interactions which are not represented in other large scale models, highlight the importance of groundwater as a mediator of system response to change, and the need to understand and incorporate subsurface storage dynamics into long term watershed projections' is fully justified.

If I were to recommend any changes it would be to encourage the authors to consider exploring the implications of their results for future modelling studies. This could be included in a slightly extended discussion. Specifically, at L105-108, it is noted that despite a uniformly warm perturbation resulting changes in PET are spatially variable due to nonlinear relationships between T, specific humidity and PET, and at L210 to 216 it is noted that sub-regional scale heterogeneity in change in groundwater storage illustrated in Figure 4 is influenced by topography and hydraulic conductivity of the subsurface. Given the degree of system heterogeneity, the degree of nonlinearity in system response to a simple single monotonic forcing, and the computational demands of the current study, what are the challenges and next steps for modelling fully-linked groundwater/surface processes systems driven by mature change scenarios?

Specific comments

1. L14. ‘warming only exacerbates …’. Incomplete sentence, what is exacerbated?
2. L20. ’decreasing response …’. Incomplete sentence, what is showing a decreasing response?
3. L69. Formatting of ‘deg. C’ and elsewhere in the text?
4. L83. Consistency in reference to ‘Methods’ section. It is capitalised elsewhere.
5. L8. Change to ’(precipitation, P)’
6. L90. Define ‘Aridity Index (AI)’ as term used in Figure 5 later in paper.
7. L98. Define ‘ET’ – first time this abbreviation is used.
8. Figure 1 and subsequent figures containing maps. Please add some geographical reference co-ordinates to the maps, or consider including a location map in Figure 1.
9. Figure 1. The in figure caption to figure 1c. ‘ Energy lim./Water lim. Is not clear and overlaps with figure. Consider moving explanatory text to main figure caption.
10. L132. Consider citing Cuthbert et al., Nature Climate Change, 9, 137–141 (2019)
11. L146. Add space to ‘(Figure 3a-d)’
12. L155. Add ‘in’ as follows: ‘especially clear in the southeast’
13. Figures 3b-3d. The figures shows some areas in the mid-west and west with a small decline in annual total ET. Please explain.
14. Figure 3f. Please look at formatting of ‘f’ – too close to sub-figure, it needs to be consistent with other sub-figure formatting.
15. L181-182 and Figure 4. There appears to be no effect of warming on rate of storage increase during winter recharge (see year 1 in Figure 4a), but there does appear to be a systematic shortening of the length of the recharge period with warming. This could be noted and perhaps quantified in the text.
16. Figure 4a. Missing data for 1.5 deg. C scenario during year 2. Please add or explain why missing.
17. Figure 5. Why are only the first and last years of modelled results presented in these figures? I assume for clarity, but it would be helpful if it can be confirmed and noted that the desribed trends are consistent across four years of data.
18. L255, Figure caption 5. Clarification of ‘availability’ of what?
19. L265. Define LH
20. L281. Should read ‘interactions that are not represented’?
21. L308. Formatting, should read ‘documented by 27’?
22. L311-313. Please define ‘water year’ in the context of the current study.

Reviewer #4 (Remarks to the Author):

In addition to some editorial suggestions on the manuscript I would say I have two recommendations.

First, it would be important to not only report the magnitude of the increased ET and loss of gw recharge across the US, but to include the loss as a percent of the existing recharge rate. When you do this you will find that it it the High Plains and Rocky Mountains that receive the largest percent loss of their recharge. These areas already have lower flowing streams and their base flows may be cut in half. Contrarily the Eastern US may have higher magnitude increases in ET, but their baseflows may only decrease by less than 20 percent.

Second, there are some processes that may counter the effect you clearly demonstrate. The major one that comes to mind is that soil infiltration rates decrease with temperatures and vice versa. Thus during warming climate the soils may allow for additional recharge, providing with water for ET that will not be a loss seen at the water table. It is worth mentioning this toward the end of the paper.

Reviewer #1 (Remarks to the Author):
Review of "Evapotranspiration depletes groundwater under warming" by Condon et al.

Recommendation: Reject, encourage to resubmit

Overall comments:
This article addresses an important topic, namely the effects of regional warming on evapotranspiration and potentially resulting groundwater depletion. However, there is a fundamental flaw in the article:

To simulate warming, the study authors use an offline hydrological model, which they drive with what they call a "uniform pseudo-warming scenario". What they do is to use observations and add respectively, 1.5°, 2° or 4°C on the observed temperatures that are used to drive the model. Unfortunately, these scenarios are not realistic for several reasons:

- Regional warming can be much larger than the global warming. This is clear from several assessments. In many regions, warming may be up to 2-3 times larger than in global mean. The authors cite the IPCC special report on 1.5°C warming, but as can be seen in figures of the report, the mean warming in the United States when global warming reaches 1.5°C is substantially larger, and even more so for extremes (See chapter 3 of the report, Figs. 3.3 and 3.4: https://www.ipcc.ch/site/assets/uploads/sites/2/2019/05/SR15_Chapter3_Low_Res.pdf).

Response:
We thank the reviewer for recognizing the important contribution our work represents. While we agree with the reviewer that regional warming, specifically over the land is projected to be higher than the global average, and that there can be spatial differences in warming response. However, we still feel that our range of temperatures (i.e. 1.5 to 4.0 ℃) is representative of the range of projected warming for the US. We have copied Figure 3.3 from the IPCC report below for reference. This shows that for a global warming scenario of 1.5 ℃, the mean warming over the majority of our study area is 1.5 to 2.0 ℃, with a small area in the inter mountain west warming 2.0 to 3.0 ℃. Similarly, for the global 2.0 ℃ warming scenario the majority of our study area increases 2.0 to 3.0 ℃. This increase in temperature range is well within our climate perturbations of 1.5 to 4 ℃. We also want to emphasize that our simulations were constructed as numerical experiments to study the potential process interactions in the terrestrial hydrologic cycle under increased temperature, not climate projections.

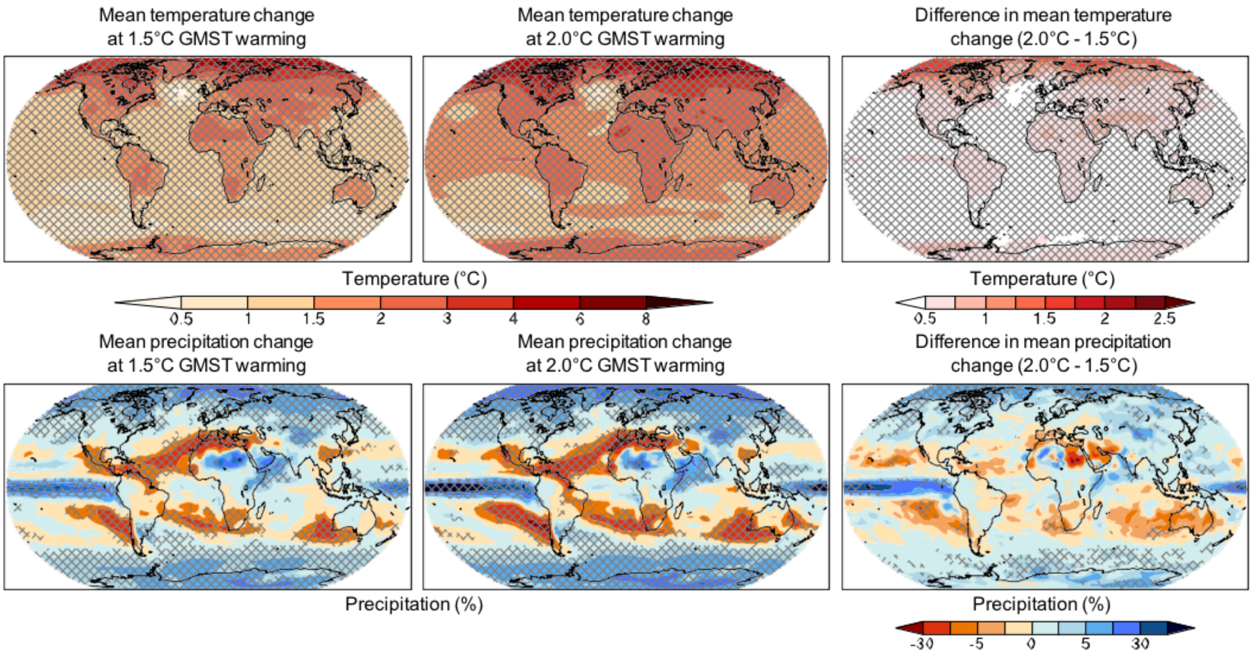

Figure 3.3 | Projected changes in mean temperature (top) and mean precipitation (bottom) at 1.5°C (left) and 2°C (middle) of global warming compared to the pre-industrial period (1861–1880), and the difference between 1.5°C and 2°C of global warming (right). Cross-hatching highlights areas where at least two-thirds of the models agree on the sign of change as a measure of robustness (18 or more out of 26). Values were assessed from the transient response over a 10-year period at a given warming level, based on Representative Concentration Pathway (RCP)8.5 Coupled Model Intercomparison Project Phase 5 (CMIP5) model simulations (adapted from Seneviratne et al., 2016 and Wartenburger et al., 2017, see Supplementary Material 3.SM.2 for more details). Note that the responses at 1.5°C of global warming are similar for RCP26 simulations (see Supplementary Material 3.SM.2). Differences compared to 1°C of global warming are provided in the Supplementary Material 3.SM.2.

In addition to the IPCC report, our choice of temperature perturbations is also based off of the national assessments provided in the US based Climate Science Special Report: Forth National Assessment[1] (Figure 6.7 from this report is copied below and the report is also now cited in the manuscript). This report projects that annual average temperatures in the US will increase by 1.4 ℃ by midcentury across all RCP scenarios relative to 1975-2005 mean temperatures. By the end of the century, these projections increase to 1.6-4.1 ℃ for the RCP 4.5 scenario, and 3.2-6.6 ℃ in the higher RCP8.5 scenario.

[redacted]

Figure 6.7 (Figure and caption copied from the Climate Science Special Report[1]): Projected changes in annual average temperatures (°F). Changes are the difference between the average for mid-century (2036–2065; top) or late-century (2070-2099, bottom) and the average for near-present (1976–2005). Each map depicts the weighted multimodel mean. Increases are statistically significant in all areas (that is, more than 50% of the models show a statistically significant change, and more than 67% agree on the sign of the change). (Figure source: CICS-NC and NOAA NCEI).

Our chosen range of 1.5 to 4 ℃ covers the potential temperature increase range in the US for mid-century projections and most of the projected range for end of century projections and therefore we feel it is a reasonable temperature range to use.

We also acknowledge that warming over land may be larger in the case of extreme events. This is explicitly stated in the referenced IPCC report as follows: "For differences in regional temperature ***extremes*** *at a mean global warming of 1.5°C versus 2°C, that is, a difference of 0.5°C in global warming, this implies differences of as much as 1°C–1.5°C in some locations, which are two to three times larger than the differences in global mean temperature." However, the emphasis from the IPCC report and specifically Fig 3.4 is for extremes (change in hottest days and coldest nights). While we believe to be an important research topic is not the focus of this work. As stated at L71-72 in our manuscript the purpose of this work is "... to directly evaluate the sensitivity of the terrestrial hydrologic system to varying degrees of warming, separate from variability and uncertainty*

in warming." Therefore, we are focused on long term average warming trends and not extreme events and changes in temporal variability. Nevertheless, we agree with the reviewer that the distinction between mean temperature perturbations and the extreme conditions is important to make. We have re-written this part of the discussion in the manuscript and elaborated on these important points as follows:

> *We compare a historical baseline climate scenario with three perturbed scenarios with uniform warming of 1.5, 2, and 4°C. The purpose of the uniform so-called, pseudo-warming[2] perturbations applied here is to directly evaluate the sensitivity of the terrestrial hydrologic system to varying degrees of warming in a series of controlled experiments. We acknowledge that climate projections show spatially variable warming trends, and temporal variability in warming especially with extreme events[3], our goal is not to capture this variability but to evaluate response to a range of long-term warming possibilities.*
>
> *The three warming scenarios selected here span the range of projections for the CONUS over the 21st century: 1.5°C is the expected warming by the middle of the 21st century if current warming trends continue[4], 2 and 4°C further provide a range of possible warming scenarios by the end of the 21st century[3]. Some parts of the country are projected to warm faster or slower than these national averages. For example, the Northeast is projected to warm faster than the Southwest[3,5]. Therefore, our 4°C scenario may be a shorter-term likelihood for some parts of the country than others. The three temperatures chosen here are not intended to be national projections for a specific time period. We seek to quantify the response to warming separate from considerations of variability and uncertainty in warming projections therefore these values were chosen to represent a range of warming that is reasonable across CONUS.*

- The authors seem to misunderstand a "1.5°C warmer climate" as a climate in which regional temperature is warming uniformly by 1.5°C. As highlighted above, this is a fundamental misunderstanding. This assumption is reiterated in the text of the article when referring to the IPCC SR15 report: "1.5 deg. C is the expected warming by the middle of the 21st century if current warming trends continue. 2 and 4 deg. C are the projected warming by the end of the 21st century given low and high emissions scenarios respectively". Obviously, these sentences are only true for the global mean warming, not for the regional warming. Overall, land warming is always higher than the global mean warming, because land warms more than the oceans.

Response:

Contrary to the reviewers comment we do not think that a 1.5°C warmer global climate indicates spatially uniform warming, and in fact we had stated this at L68-70 in the original manuscript as follows *(Directly above the sentence that the reviewer referenced here):*

> While climate projections show spatially variable warming trends, the purpose of the uniform so-called, pseudo-warming[24] perturbations applied here is to directly evaluate the sensitivity of the terrestrial hydrologic system to varying degrees of warming, separate from variability and uncertainty in warming projections.

It is unfortunate that the reviewer missed this during their review (this was very clearly articulated by the other reviewers who cited this as a strength of the work). We also note from the United States Climate Science Special Report[5] that the estimated mean temperature change from region to region within our study domain is approximately 0.9°F (0.5°C) for mid-century projections and less than 1.8°F (1°C) for end of century projections. The table presented below is Table 6.4 from the report and states the mean temperature changes in Fahrenheit (Note the slightly high temperature change

from the 1.4°C stated earlier above. This is because of a slightly later mid-century time period shown in the table). Again, this does not account for extremes, which is where most of the variability occurs but is also out of the scope of this work.

NCA Region	RCP4.5 Mid-Century (2036–2065)	RCP8.5 Mid-Century (2036–2065)	RCP4.5 Late-Century (2071–2100)	RCP8.5 Late-Century (2071–2100)
Northeast	3.98°F	5.09°F	5.27°F	9.11°F
Southeast	3.40°F	4.30°F	4.43°F	7.72°F
Midwest	4.21°F	5.29°F	5.57°F	9.49°F
Great Plains North	4.05°F	5.10°F	5.44°F	9.37°F
Great Plains South	3.62°F	4.61°F	4.78°F	8.44°F
Southwest	3.72°F	4.80°F	4.93°F	8.65°F
Northwest	3.66°F	4.67°F	4.99°F	8.51°F

Table 6.4 (Table and caption copied form Vose et al. 2017). Projected changes in annual average temperature (°F) for each National Climate Assessment region in the contiguous United States. Changes are the difference between the average for mid-century (2036–2065) or late-century (2071–2100) and the average for near-present (1976–2005) under the higher scenario (RCP8.5) and a lower scenario (RCP4.5). Estimates are derived from 32 climate models that were statistically downscaled using the Localized Constructed Analogs technique. Increases are statistically significant in all areas (that is, more than 50% of the models show a statistically significant change, and more than 67% agree on the sign of the change)[5].

We bring this table up not to indicate that regional differences are insignificant, but rather show that the regional differences are well within our range of climate perturbations. Here we should again stress as stated in Lines 58-65 of the original manuscript that our goal is to systematically isolate the role of the subsurface in watershed response to varying levels of warming, and not to project future groundwater states (which would require spatially and temporally varying projections of warming as well as other variables such as precipitation and human water use). The purpose of using a consistent warming range across our domain is to show for every location in the domain the sensitivity to a range of warming, rather than project what the future warming in these locations will be.

By systematically applying a consistent warming in space and time we are able to isolate how the hydrologic response to that warming will vary spatially as a function of subsurface hydrology. Our goal was to demonstrate how the subsurface can mitigate or accelerate response to a warming perturbation as this has not been previously demonstrated in continental scale hydrologic simulations. To do that we needed to isolate this variable from the other well established spatial and temporal climate change patterns. This is intended to isolate the role groundwater plays as a regional mediator of hydrologic change, and how sustained warming of any magnitude results in early changes in groundwaters ability to mediate hydrologic changes. We feel this is an important and novel scientific insight. Therefore, we completely agree with the reviewer that our choice of a temporally uniform temperature warming scenario does not represent a true forecast of water resources. However, we think our approach is valuable because it provides insight to the general behavior of groundwater as the climate warms. We argue that our pseudo-warming approach is not a limitation of our study but a necessary experimental design to isolate a set of critical hydrological system functions that has not been thoroughly explored to date. If we implemented more complex spatially and temporally varying forcings then our results would instead highlight the spatial and

temporal variability in climate projections which have already been established through GCM simulations. We agree with the reviewer that this is a critical point to our experimental setup and that it may not have come across clearly enough in the original manuscript. We copied our revised description in the response above and we hope that this will address the noted concerns.

- In addition, a uniform temperature anomaly also ignores further geographical or temporal variations in warming. In the highlighted figures from the IPCC SR15 report, it can be seen that some regions of the US warm more than others. In addition, the projected changes in temperature are also varying depending on the season.

Response:
Please refer to our earlier detailed responses. We understand the reviewers concern and we fully agree that global temperature changes of 1.5°C, 2°C and 4°C are not an indication that this level of warming will be observed everywhere all the time. We agree that projections show both spatial and temporal variability. We intentionally designed this study as a sensitivity analysis so that we could isolate the role of subsurface storage in watershed response from the significant spatial and temporal variability in projections of future temperatures. This allows us to compare across locations and present novel results highlighting how groundwater changes system response. However, our results are not intended to be a projection of future water availability because we have made these significant simplifications to the projections changes in temperature, and also because we did not include changes in precipitation.   We hope that our response above, in addition to the clarifications we have added to the text, will (1) demonstrate that our choice of temperature range is reasonable for our study domain and (2) make it more clear to readers that what we are presenting here is a sensitivity analysis and not a groundwater projection.

For these reasons, I can unfortunately no recommend publication of this article in Nature Communications. However, the topic is certainly interesting. If the authors could drive their simulations with realistic changes in temperature at 1.5°C, 2°C and 4°C global warming, I would recommend to reconsider publication of this article in Nature Communications.

Response:
We thank the reviewer for their consideration of our work and hope that they will consider our rationale for our experimental setup and our revised text.

Reviewer #3 (Remarks to the Author):

General comments

The authors have used ParFlow-CLM, a model that has previously been documented and calibrated across the study domain (N America), to 'evaluate the impact of increased temperature due to climate change on evapotranspiration and groundwater depth'. The authors explain that the ParFlow-CLM model has been used as it explicitly represents lateral groundwater flow and interactions between groundwater and surface water at high spatial resolutions in a manner that is not usually captured by GCMs and LCMs. The model simulations were computationally intense which clearly placed limits on the scenarios that could be considered. Nonetheless, the model experiments adequately address the authors' research question.

Condon et al. compare the effect of three uniform warming (or 'pseudo- warming') scenarios applied to a baseline temperature, on groundwater storage, where the warming scenarios span a range of climate change projections for N America. Each simulation uses a single year of historical meteorological forcing repeated for a period of four years: as the authors say 'to control for interannual variability'. It is shown that, in the absence of other change factors, warming reduces groundwater storage, but in a non-trivial manner. Water limited systems become more arid, but in doing so become less sensitive to continued or increased warming. While in less arid areas warming drives groundwater storage losses to support increased evapotranspiration where shallow groundwater is available. However, sustained warming in this context leads to reduced groundwater storage and 'decreasing response to additional warming'.

I enjoyed reading the paper - it is novel and should be of broad interest. It's well written, effectively articulates why the work has been undertaken and the importance of understanding the effect of warming on groundwater storage. The modelling methodology and underlying assumptions are sound, and the results clearly described. The overarching conclusion, that 'nonlinear interactions which are not represented in other large scale models, highlight the importance of groundwater as a mediator of system response to change, and the need to understand and incorporate subsurface storage dynamics into long term watershed projections' is fully justified.

If I were to recommend any changes it would be to encourage the authors to consider exploring the implications of their results for future modelling studies. This could be included in a slightly extended discussion. Specifically, at L105-108, it is noted that despite a uniformly warm perturbation resulting changes in PET are spatially variable due to nonlinear relationships between T, specific humidity and PET, and at L210 to 216 it is noted that sub-regional scale heterogeneity in change in groundwater storage illustrated in Figure 4 is influenced by topography and hydraulic conductivity of the subsurface. Given the degree of system heterogeneity, the degree of nonlinearity in system response to a single monotonic forcing, and the computational demands of the current study what are the challenges and next steps for modeling fully-linked groundwater/surface process systems driven by mature climate change scenarios?
Response:
We thank the reviewer for their careful consideration of our work and for acknowledging the computational demands of this approach. The reviewer is correct that this places a limitation on the number of simulations we can run and was a consideration in the design of our experiment. Also, as the reviewer notes, the goal of this intensive modeling work is to quantify potential impacts of nonlinear groundwater interactions that are missing from the tools we most commonly use to make future projections. While many recent studies note the need for better groundwater representation in large scale models, there has been very little research to quantify how groundwater and surface water interactions will evolving in warming conditions. This work, which isolates how groundwater

will affect the system under the uniform pseudo-warming conditions isolates this single system component from all of the other drivers of hydrologic change. We greatly appreciate the reviewer's positive comments and their acknowledgment of the contribution we are trying to make. We appreciate the suggestion with respect to discussion and have added the following text:

> *The nonlinear interactions outlined here are not currently represented in other large-scale models. The sensitivity of hydrologic response to shallow groundwater configuration demonstrated here highlights the importance of groundwater as a mediator of system response to change, and the need to understand and incorporate subsurface storage dynamics into long term watershed projections, especially as climate change is expected to bring about increased extremes, (i.e. not just extreme temperature, but also precipitation). Understanding the role of groundwater as an underlying system mediator to persistent temperature increases is key to understanding how the overall system will respond to these extreme events. Dynamic groundwater interactions will likely affect ecosystem productivity[6] or conversely be affected by warming induced changes to hydraulic conductivity[7,8], both of which were not considered and may counteract some of the sensitivity found here. Never-the-less, the integrated modelling approach used here demonstrates that it is technically feasible to simulate variably saturated lateral groundwater flow at high resolution across large domains; however, this remains a very computationally intensive modeling approach and global models and multi-decadal ensemble simulations are not yet feasible. Future work should focus on decreasing the computational demands of fully integrated models and using existing large-scale integrated models to validate the groundwater surface water interactions of global earth systems models as new groundwater formulations are implemented in these tools*.

Specific comments

1. L14. 'warming only exacerbates …'. Incomplete sentence, what is exacerbated? *Corrected: Changed to 'warming only exacerbates water stress'*

2. L20. 'decreasing response …'. Incomplete sentence, what is showing a decreasing response? *Corrected: Changed to 'decreasing watershed response'*

3. L69. Formatting of 'deg. C' and elsewhere in the text? *This has been changed to '°C' throughout the text.*

4. L83. Consistency in reference to 'Methods' section. It is capitalised elsewhere. *Corrected: This has been capitalized here and checked for consistency in the rest of the manuscript.*

5. L8. Change to '(precipitation, P)' *Changed.*

6. L90. Define 'Aridity Index (AI)' as term used in Figure 5 later in paper. *We have added the following definition: "Aridity measures the relative balance between water demand (calculated here as potential evapotranspiration, PET) and water supply (precipitation, P). The Aridity Index (AI, PET/P) can be used to characterize the water and energy drivers of hydrologic systems."*

7. L98. Define 'ET' – first time this abbreviation is used. *This has been added.*

8. Figure 1 and subsequent figures containing maps. Please add some geographical reference co-ordinates to the maps, or consider including a location map in Figure 1. *We appreciate the suggestion and have added a map of the US showing the domain area to Figure 1 for reference.*

9. Figure 1. The in figure caption to figure 1c. ‘ Energy lim./Water lim. Is not clear and overlaps with figure. Consider moving explanatory text to main figure caption. *This figure has been modified so that the water and energy limitation lines are now inside the figure and don’t appear as a caption. We also extended the vertical line in the figure to try to make this more clear.*

10. L132. Consider citing Cuthbert et al., Nature Climate Change, 9, 137–141 (2019) *We have added that citation here.*

11. L146. Add space to ‘(Figure 3a-d)’ *This has been added.*

12. L155. Add ‘in’ as follows: ‘especially clear in the southeast’ *This has been added.*

13. Figures 3b-3d. The figures shows some areas in the mid-west and west with a small decline in annual total ET. Please explain. *This is caused by an expansion of the water limited season and persistent drying which decreases water availability. We have clarified this in the revised manuscript as follows:*

> *In some parts of the west, there are actually small declines in ET with warming as the water limited portion of the year expands and the system dries further limiting water availability in these already dry locations*

14. Figure 3f. Please look at formatting of ‘f’ – too close to sub-figure, it needs to be consistent with other sub-figure formatting. *This has been shifted in the revised manuscript to make it consistent.*

15. L181-182 and Figure 4. There appears to be no effect of warming on rate of storage increase during winter recharge (see year 1 in Figure 4a), but there does appear to be a systematic shortening of the length of the recharge period with warming. This could be noted and perhaps quantified in the text. *These are interesting points. Actually, the rate of storage increase (calculated as the max storage minus they day 1 storage for every year) does go up over the course of the scenario but the length of the recharge period (calculated as the date of the max storage) stays relatively constant. We have added the following quantitative discussion of this point to the revised manuscript:*

> The annual recharge (i.e. the storage difference from the first day of each year to the day of peak storage) increases by 16%, 24% and 49% for the 1.5, 2, and 4°C cases respectively comparing between the first and last years of each scenario.

16. Figure 4a. Missing data for 1.5 deg. C scenario during year 2. Please add or explain why missing. *This has been corrected in the revised manuscript the new Figure 4 has this line included.*

17. Figure 5. Why are only the first and last years of modelled results presented in these figures? I assume for clarity, but it would be helpful if it can be confirmed and noted that the desribed trends are consistent across four years of data. *Yes, the shifting is easiest to observe comparing across the two end years of the simulation and including all four years on the plot made if very difficult to interpret so we decided to focus on the first and last years for clarity here. This is a good point though and we have added the following clarification to the manuscript:*

> *Similar, but less pronounced shifting, can also be seen between intermediate simulation years, we present only the first and last years here for clarity*

18. L255, Figure caption 5. Clarification of ‘availability’ of what? *This has been clarified as ‘water availability’*

19. L265. Define LH *This should have been ET. It has been corrected in the revised manuscript.*

20. L281. Should read ‘interactions that are not represented’? *This has been corrected*

21. L308. Formatting, should read ‘documented by 27’? *This has been corrected*

22. L311-313. Please define ‘water year’ in the context of the current study. *The dates of the simulation have been clarified here. Also it was incorrectly listed as WY 1984 instead of 1985 in the original manuscript and this has now been corrected.*

Reviewer #4 (Remarks to the Author):

In addition to some editorial suggestions on the manuscript I would say I have two recommendations.

First, it would be important to not only report the magnitude of the increased ET and loss of gw recharge across the US, but to include the loss as a percent of the existing recharge rate. When you do this you will find that it it the High Plains and Rocky Mountains that receive the largest percent loss of their recharge. These areas already have lower flowing streams and their base flows may be cut in half. Contrarily the Eastern US may have higher magnitude increases in ET, but their baseflows may only decrease by less than 20 percent.

Response:

We appreciate the suggestion and agree that these relative calculations are a great way to put our work in context. We don't calculate baseflow directly in this study but we have included two tables below the changes in ET and storage relative to the baseline ET and the baseline potential recharge (Precipitation – ET). As, shown below actually the relative changes in ET and storage are still largest in the Eastern part of the domain even though those areas do have larger baseline ET and potential recharge. However, we agree with the reviewer that in the more water limited western portions of the domain small changes in recharge and baseflow can have larger consequences on water management because these systems are already so water limited. We think this is an important point to make and have added to the discussion in the revised manuscript as follows:

> *Potential recharge (calculated as precipitation minus ET) in the western more arid basins decreases by 12-15% relative to the Baseline scenario. This study focuses on storage connections to ET dynamics so the changes to recharge are not discussed in detail here. However, it should be noted that groundwater and baseflow are important to the water supply systems across the western US. Although, the relative increases in ET and declines in recharge are smaller in the west than the east; the heavily managed and often overallocated systems in the west are more sensitive to small changes in water supply.*

Table 1: Changes in ET and storage calculated relative to the baseline ET for Year 4 of the simulation

	Simulated Year 4 Annual Average ET [mm]				ET % Difference (ET Difference/Baseline ET)			Storage % Difference (ET Difference/Baseline ET)		
**Region**	**Baseline**	**1.5 deg C**	**2 deg C**	**4 deg C**	**1.5 deg C**	**2 deg C**	**4 deg C**	**1.5 deg C**	**2 deg C**	**4 deg C**
Great Basin	237.1	243.6	245.2	251.2	3%	3%	6%	-4.5%	-7.2%	-15.8%
Lower Colorado	294.0	301.3	303.3	310.2	2%	3%	5%	-4.9%	-6.7%	-12.6%
Upper Colorado	297.4	310.3	313.6	326.5	4%	5%	10%	-4.8%	-7.9%	-16.5%
Rio Grande	402.2	413.5	416.6	427.9	3%	4%	6%	-4.7%	-6.9%	-13.6%
Pac. Northwest	346.4	372.3	378.3	399.5	7%	9%	15%	-5.2%	-9.1%	-18.5%
Missouri	417.8	439.5	445.0	463.8	5%	7%	11%	-4.7%	-6.5%	-12.2%
Arkansas	618.7	664.9	677.3	717.6	7%	9%	16%	-2.1%	-2.8%	-4.8%
Lower Mississippi	917.8	1024.4	1051.9	1135.9	12%	15%	24%	-3.1%	-4.0%	-6.6%
Upper Mississippi	607.9	672.4	689.9	746.1	11%	13%	23%	-3.6%	-4.7%	-8.7%
Tennessee	818.5	928.0	956.5	1043.9	13%	17%	28%	-4.8%	-6.4%	-12.3%
Ohio	729.1	832.4	859.8	946.4	14%	18%	30%	-5.2%	-7.1%	-14.7%

Table 2: Changes in ET and storage calculated relative to the baseline potential recharge (Precipitation-ET) for Year 4 of the simulation

	Average potential Recharge (Preicip - LH, [mm])				%change in potential Recharge]		
**Region**	**Baseline**	**1.5 deg C**	**2 deg C**	**4 deg C**	**1.5 deg C**	**2 deg C**	**4 deg C**
Great Basin	53.5	47.0	45.5	39.4	-12%	-15%	-26%
Lower Colorado	77.1	69.9	67.9	61.0	-9%	-12%	-21%
Upper Colorado	120.5	107.6	104.3	91.4	-11%	-13%	-24%
Rio Grande	100.4	89.0	86.0	74.6	-11%	-14%	-26%
Pac. Northwest	178.4	152.5	146.5	125.2	-15%	-18%	-30%
Missouri	116.3	94.6	89.1	70.3	-19%	-23%	-40%
Arkansas	347.5	301.3	288.9	248.6	-13%	-17%	-28%
Lower Mississippi	486.2	379.5	352.1	268.0	-22%	-28%	-45%
Upper Mississippi	340.5	276.0	258.5	202.3	-19%	-24%	-41%
Tennessee	416.1	306.6	278.1	190.7	-26%	-33%	-54%
Ohio	374.9	271.7	244.2	157.7	-28%	-35%	-58%

Second, there are some processes that may counter the effect you clearly demonstrate. The major one that comes to mind is that soil infiltration rates decrease with temperatures and vice versa. Thus during warming climate the soils may allow for additional recharge, providing with water for ET that will not be a loss seen at the water table. It is worth mentioning this toward the end of the paper.

Response:

We agree with the reviewer that this is an important consideration that should be considered further in future work. In response to this and the following comment the reviewer left in the text we added the following to the end of the manuscript:

> Dynamic groundwater interactions will likely affect ecosystem productivity[6] or conversely be affected by warming induced changes to hydraulic conductivity[7,8], both of which were not considered and may counteract some of the sensitivity found here.

Editorial Suggestion copied from the text:

You should mention factors that may counter your assumptions. There are studies that suggest plant stomas tend to close, reducing ET, as CO2 levels increase. Also studies show that surface runoff is a function of temperature, not just because less ground is frozen but because soil properties such as capillary forces are temperature dependent leading to slower infiltration rates with colder water. Thus during warmer weather infiltration and recharge are higher given the same precipitation rates. A recent study found this to be true across the summer when comparing runoff in cold months versus warm months.

Constantz J, 1982, Temperature dependence of unsaturated hydraulic conductivity of two soils 1. Soil Soc. Am. J. 46 (3), 466-470.

Reitz M., Sanford W., 2019, Estimating quick-flow runoff at the monthly time scale for the conterminous United States, J. of Hydrology 573, 841-854.

Response:

See response above. We agree with the reviewer that these are important caveats to note and have added the following sentence to the end of the manuscript with these citations to make this point.

Dynamic groundwater interactions will likely affect ecosystem productivity[6] or conversely be affected by warming induced changes to hydraulic conductivity[7,8], both of which were not considered and may counteract some of the sensitivity found here.

References

1 Usgcrp. Climate Science Special Report: Fourth National Climate Assessment, Volume I. 470 (U.S. Global Change Research Program, Washington, DC, USA, 2017).

2 Rasmussen, R. *et al.* High-Resolution Coupled Climate Runoff Simulations of Seasonal Snowfall over Colorado: A Process Study of Current and Warmer Climate. **24**, 3015-3048, doi:10.1175/2010jcli3985.1 (2011).

3 Hayhoe, K. *et al.* Our Changing Climate. In Impacts, Risks, and Adaptation in the United States: Fourth National Climate
Assessment, Volume II (U.S. Global Change Research Program, Washington, DC, USA, 2018).

4 IPCC. Summary for Policymakers. In: Global warming of 1.5°C. An IPCC Special Report on the impacts of global warming of 1.5°C above pre-industrial levels and related global greenhouse gas emission pathways, in the context of strengthening the global response to the threat of climate change, sustainable development, and efforts to eradicate poverty. (World Meteorological Organization, Geneva, Switzerland, 2018).

5 Vose, R. S., Easterling, D. R., Kunkel, K. E., LeGrande, A. N. & Wehner, M. F. in *Climate Science Special Report: Fourth National Climate Assessment, Volume I* (eds D. J. Wuebbles *et al.*) 185-206 (U.S. Global Change Research Program, 2017).

6 Burkett, V. R. *et al.* Nonlinear dynamics in ecosystem response to climatic change: Case studies and policy implications. *Ecological Complexity* **2**, 357-394, doi:https://doi.org/10.1016/j.ecocom.2005.04.010 (2005).

7 Constantz, J. Temperature Dependence of Unsaturated Hydraulic Conductivity of Two Soils 1. *Soil Science Society of America Journal* **46**, 466-470 (1982).

8 Reitz, M. & Sanford, W. E. Estimating quick-flow runoff at the monthly timescale for the conterminous United States. *Journal of Hydrology* **573**, 841-854, doi:10.1016/j.jhydrol.2019.04.010 (2019).

Reviewers' comments:

Reviewer #1 (Remarks to the Author):

Review of Condon et al. (resubmitted)

Recommendation: Reject

General comments:

The authors have still not taken my comments into account. They recognise in their answer that their "pseudo-warming" experiment is not realistic and very different from actual climate projections, but the article has not been adapted. For instance, the manuscript includes the following sentences describing the set-up of the experiments:

"The three warming scenarios selected here span the range of projections for the CONUS over the 21st century; 1.5°C is the expected warming by the middle of the 21st century if current warming trends continue, 2 and 4°C further provide a range of possible warming scenarios by the end of the 21st century." This is too little context. The authors could for instance have provided actual analyses of climate projections for comparison with their "pseudo-warming experiments".

Another issue (which I had not noted in my first review) is that it appears that the authors do not consider possible direct effects of CO_2 on evapotranspiration. Because enhanced CO_2 could increase water-use efficiency of plants, this can reduce evapotranspiration under a warmer climate (e.g. Lemordant et al. 2016).

The study seems too conceptual and far from a realistic set-up. It is based on a single model, while it is well known that different hydrological models can present very different responses to climate forcing (e.g. Prudhomme et al. 2014). It also does not include changes in radiation, atmospheric humidity or precipitation, which would all be modified under realistic climate-change projections.

Based on these various points, I cannot recommend publication of this manuscript in Nature Communications. The article would be suitable for a disciplinary journal (e.g. ERL, GRL). But this study is not suitable at present for a high-impact journal, because it is not clear enough to which extent the results would be robust if a different experimental set-up or hydrological model were used.

References:

- Lemordant, L., et al. 2016: Modification of land-atmosphere interactions by CO_2 effects: Implications for summer dryness and heat wave amplitude. Geophys. Res. Lett., 43, doi:10.1002/2016GL069896.

- Prudhomme, C., et al. 2014: Hydrological droughts in the 21st century, hotspots and uncertainties from a global multimodel ensemble experiment. PNAS, www.pnas.org/cgi/doi/10.1073/pnas.1222473110

Reviewer #3 (Remarks to the Author):

Many thanks for addressing in detail all my comments (reviewer #3). I'm happy with all your proposed changes.

John Bloomfield

Reviewer #4 (Remarks to the Author):

The authors have made a conscientious effort to responds to the three reviewers comments on their manuscript. I agree with the authors concerning the first reviewer's comments, which where the most critical of the three. The reviewer argues that the authors make a grave error by treating all of the conterminous US as having increasing temperatures that are constant in space, not taking into account N-S or E-W trends across the continent, which might be substantial, and it is implied this is a misunderstanding of the authors and limits the usefulness of the results. But my thought before even reading the authors' response was that they knew this to be the case but were not trying to simulate specific scenarios projected by the climate models, but were trying to look at a more general response of the hydrology to increasing temperatures. When taken in this context their numerical experiments give meaningful results. And indeed the author's response indicated that was their intent to show all along.

The second and third reviewer's were less critical, but offered advice for more meaningful discussions of the results, and the author's did a good job of incorporating these suggestions into the text of the manuscript--including additional tables to illustrate changes in ET as a percent of recharge.

I believe that given the changes made by the authors that this manuscript should now be accepted by the journal for publication.

Reviewer #1 (Remarks to the Author):

Review of Condon et al. (resubmitted)

Recommendation: Reject

General comments:

The authors have still not taken my comments into account. They recognise in their answer that their "pseudo-warming" experiment is not realistic and very different from actual climate projections, but the article has not been adapted. For instance, the manuscript includes the following sentences describing the set-up of the experiments:

"The three warming scenarios selected here span the range of projections for the CONUS over the 21st century; 1.5°C is the expected warming by the middle of the 21st century if current warming trends continue, 2 and 4°C further provide a range of possible warming scenarios by the end of the 21st century." This is too little context. The authors could for instance have provided actual analyses of climate projections for comparison with their "pseudo-warming experiments".
Response:
We are sorry to hear that the reviewer felt that this sentence indicated that we had not listened to their original review. We disagree with this assessment though given that we (1) added significant discussion to the original submission regarding the purpose of using our 'pseudo-warming approach at Lines 99-115 and (2) did provided references and figures based on actual analyses of IPCC projections to support this choice in our last response.

As we discussed extensively in our previous response, we chose these warming levels as representative perturbations. In our previous response we provided figures and references indicating how we decided that these temperature perturbations would be representative. More importantly we stand by our approach as a means to illuminate the fundamental interaction of groundwater processes with systematic warming and therefore, provide a critical advancement in understanding how surface water and groundwater interactions will change due to warming. Incorporating transient and heterogenous projections directly from IPCC model outputs would not yield a better understanding of the groundwater response to warming and would in fact limit our ability to isolate this critical connection. Nevertheless, we have made every effort to be transparent about this approach and the fact that we are not making projections here but evaluating sensitivity.

Finally, while the reviewer pulled out one sentence of our description in their comment above, we would like to point out that in response to their previous comments we did expand on the description of our methodology as follows.

> *We compare a historical baseline climate scenario with three perturbed scenarios with uniform warming of 1.5, 2, and 4°C. The purpose of the uniform so-called, pseudo-warming(Rasmussen et al., 2011) perturbations applied here is to directly evaluate the sensitivity of the terrestrial hydrologic system to varying degrees of warming in a series of controlled experiments. We acknowledge that climate projections show spatially variable warming trends, and temporal variability in warming especially with extreme events(Hayhoe et al., 2018), our goal is not to capture this variability but to evaluate response to a range of long-term warming possibilities.*

The three warming scenarios selected here span the range of projections for the CONUS over the 21st century: 1.5°C is the expected warming by the middle of the 21st century if current warming trends continue(IPCC, 2018), 2 and 4°C further provide a range of possible warming scenarios by the end of the 21st century(Hayhoe et al., 2018). Some parts of the country are projected to warm faster or slower than these national averages. For example, the Northeast is projected to warm faster than the Southwest(Hayhoe et al., 2018; Vose et al., 2017). Therefore, our 4°C scenario may be a shorter-term likelihood for some parts of the country than others. The three temperatures chosen here are not intended to be national projections for a specific time period. We seek to quantify the response to warming separate from considerations of variability and uncertainty in warming projections therefore these values were chosen to represent a range of warming that is reasonable across CONUS.

We think this description is transparent about the limitations and assumptions that the reviewer brought in their previous review. We thank them for those comments which we do feel improved the clarity of the manuscript.

Another issue (which I had not noted in my first review) is that it appears that the authors do not consider possible direct effects of CO2 on evapotranspiration. Because enhanced CO2 could increase water-use efficiency of plants, this can reduce evapotranspiration under a warmer climate (e.g. Lemordant et al. 2016).

Response:

The reviewer brings up an important point that echoes Reviewer 4's original comment: "You should mention factors that may counter your assumptions. There are studies that suggest plant stomas tend to close, reducing ET, as CO2 levels increase." Based on that comment from Reviewer 4 we added the following text to the original revised manuscript (Line 312):

"Dynamic groundwater interactions will likely affect ecosystem productivity(Burkett et al., 2005) or conversely be affected by warming induced changes to hydraulic conductivity(Constantz, 1982; Reitz & Sanford, 2019), both of which were not considered and may counteract some of the sensitivity found here."

We would like to point out that Reviewer 4 accepted this change and strongly endorses the revised manuscript. Still, we acknowledge the point that and agree that this point could be further highlighted. We have further revised this as follows:

Dynamic groundwater interactions will likely affect ecosystem productivity(Burkett et al., 2005) or conversely be affected by warming induced changes to hydraulic conductivity (Constantz, 1982; Reitz & Sanford, 2019). Also, CO_2 enrichment may increase water use efficiency and partly compensate for increased evaporative demand caused by warming (Guerrieri et al., 2019; Lemordant et al., 2016). These interactions were not considered in this study and may counteract some of the sensitivity found here.

Finally, we would like to stress that although this is an important consideration, the future impacts of CO_2 enrichment are still a very active research area with much uncertainty. CO_2 enrichment can have competing impacts on total plant water as increased leaf area index may counter increased water use efficiency. Furthermore, as pointed out in a recent PNAS paper: *"Multiple lines of evidence suggest that plant water-use efficiency (WUE)—the ratio of carbon assimilation to water loss—has increased in recent decades. Although rising atmospheric CO2 has been proposed as the principal cause, the underlying*

physiological mechanisms are still being debated, and implications for the global water cycle remain uncertain" (Guerrieri et al., 2019). Modeling how these small scale processes can be accurately captured at the scales we are modeling remains an active area of research and model development (Fatichi et al., 2016; Lin et al., 2015; Xu et al., 2013). Thus, while we agree that these interactions are worth mentioning we stand by our decision not to incorporate this into our controlled groundwater sensitivity analysis.

The study seems too conceptual and far from a realistic set-up. It is based on a single model, while it is well known that different hydrological models can present very different responses to climate forcing (e.g. Prudhomme et al. 2014). It also does not include changes in radiation, atmospheric humidity or precipitation, which would all be modified under realistic climate-change projections.
Response:
The referred Prudhomme et al. paper compares hydrologic projections from several global impact models (GIMs) and explicitly demonstrates how different hydrologic representations produce large outcome uncertainty based on model structure, i.e. process representation (here we should note that none of the models in the Prudhomme paper include dynamic groundwater lateral flow and interaction with surface water, the specific process focus of our manuscript). The large discrepancies between models shown by this paper precisely demonstrates why we need to improve our processed based understanding to provide better constrained and more actionable hydrologic projections.

Our work isolates, and therefore quantifiably demonstrates, the importance of dynamic groundwater interactions in watershed response to warming. Representing, and more importantly understanding, the role of storage changes in a changing climate is a consistent limitation in existing global models and critical need that has been expressed repeatedly in the literature including in high profile journals (B.E. Jiménez Cisneros et al., 2014; Humphrey et al., 2018; Jung et al., 2017; Lemordant et al., 2016; Ove Hoegh-Guldberg et al., 2018). We base our work on a single model because ours is the only model that explicitly representing these groundwater interactions across the US. We have added the following text to the revised manuscript to highlight this point more clearly and point to the potential for this type of comparison with future work:

> *While currently only one, integrated hydrologic model exists for North America, as more simulations of this type are undertaken a multi-model approach(Prudhomme et al., 2014) could be used to further study the impact of conceptual model uncertainty on response or to propagate warming projections to groundwater.*

While we have designed a controlled numerical experiment, this approach is far from conceptual. We are simulating 3D variably saturated flow across millions of square kilometers. This is the most computationally advanced hydrologic simulation currently available for the US. We are choosing a simple perturbation to control for the number of degrees of freedom in our experiment and provide tractable results that can be useful to other models that either neglect subsurface flow or rely on more conceptual groundwater representations. This type of controlled numerical experiment is a very common way that the research community is working through complex interactions. As just one example, the Lemordant et al. (2016) paper the reviewer cited above takes the same approach; holding other variables constant while systemically changing CO_2 levels to, *"to estimate the sole impact of CO2 physiological effects" (Lemordant et al., 2016).*

We recognize the reviewer's fundamental disagreement of our work, in that by not representing all possible processes that contribute hydrological conditions we are unable to produce a valid hydrologic projection as a result of climate change. However, what the reviewer has chosen to neglect is that process understanding is foundational to scientific knowledge and work such as ours that aims to understand the underlying processes are specifically relevant and of particular interest to the climate community for the reasons pointed out by the Prudhomme et al., 2014 paper.

Based on these various points, I cannot recommend publication of this manuscript in Nature Communications. The article would be suitable for a disciplinary journal (e.g. ERL, GRL). But this study is not suitable at present for a high-impact journal, because it is not clear enough to which extent the results would be robust if a different experimental set-up or hydrological model were used.
Response*:* We are sorry that the reviewer does not appreciate the contributions of this work and the significant and unprecedented nature of the simulations we developed here. As noted above, there are no other hydrologic models simulating the integrated processes we consider over the Continental US. We are using a rigorous and computationally intensive approach to quantify interactions based on our best scientific understanding of subsurface flow and groundwater surface water interactions. This is valuable to a broad community precisely because other models are not incorporating these processes and our global community is struggling to understand what the role of storage will be in a warmer climate.

References:

- Lemordant, L., et al. 2016: Modification of land-atmosphere interactions by CO2 effects: Implications for summer dryness and heat wave amplitude. Geophys. Res. Lett., 43, doi:10.1002/2016GL069896.

- Prudhomme, C., et al. 2014: Hydrological droughts in the 21st century, hotspots and uncertainties from a global multimodel ensemble experiment. PNAS, www.pnas.org/cgi/doi/10.1073/pnas.1222473110

Reviewer #3 (Remarks to the Author):

Many thanks for addressing in detail all my comments (reviewer #3). I'm happy with all your proposed changes.

John Bloomfield

Response:
Thank, you for your suggestions John. We think they improved the revised manuscript and we are happy that you agree.

Reviewer #4 (Remarks to the Author):

The authors have made a conscientious effort to responds to the three reviewers comments on their manuscript. I agree with the authors concerning the first reviewer's comments, which where the most critical of the three. The reviewer argues that the authors make a grave error by treating all of the conterminous US as having increasing temperatures that are constant in space, not taking into account N-S or E-W trends across the continent, which might be substantial, and it is implied this is a misunderstanding of the authors and limits the usefulness of the results. But my thought before even reading the authors' response was that they knew this to be the case but were not trying to simulate specific scenarios projected by the climate models, but were trying to look at a more general response of the hydrology to increasing temperatures. When taken in this context their numerical experiments give meaningful results. And indeed the author's response indicated that was their
intent to show all along.

The second and third reviewer's were less critical, but offered advice for more meaningful discussions of the results, and the author's did a good job of incorporating these suggestions into the text of the manuscript--including additional tables to illustrate changes in ET as a percent of recharge.

I believe that given the changes made by the authors that this manuscript should now be accepted by the journal for publication.

Response:
Thank, you for the thoughtful consideration of our work. We agree with your assessment and have made every effort to be transparent about our methodology and assumptions at every step of the way. We appreciated all of the suggestions we received in the first round and we think they improved the manuscript significantly.

References

B.E. Jiménez Cisneros, Oki, T., Arnell, N. W., Benito, G., Cogley, J. G., Petra, D., et al. (2014). *Freshwater resources. In: Climate Change 2014: Impacts, Adaptation, and Vulnerability. Part A: Global and*

Sectoral Aspects. Contribution of Working Group II to the Fifth Assessment Report of the Intergovernmental

Panel on Climate Change. Retrieved from Cambridge, United Kingdom and New York, NY USA:

Burkett, V. R., Wilcox, D. A., Stottlemyer, R., Barrow, W., Fagre, D., Baron, J., et al. (2005). Nonlinear dynamics in ecosystem response to climatic change: Case studies and policy implications. *Ecological Complexity, 2*(4), 357-394. http://www.sciencedirect.com/science/article/pii/S1476945X05000334

Constantz, J. (1982). Temperature Dependence of Unsaturated Hydraulic Conductivity of Two Soils 1. *Soil Science Society of America Journal, 46*(3), 466-470.

Fatichi, S., Pappas, C., & Ivanov, V. Y. (2016). Modeling plant-water interactions: an ecohydrological overview from the cell to the global scale. *Wiley Interdisciplinary Reviews: Water, 3*(3), 327-368. http://dx.doi.org/10.1002/wat2.1125

Guerrieri, R., Belmecheri, S., Ollinger, S. V., Asbjornsen, H., Jennings, K., Xiao, J., et al. (2019). Disentangling the role of photosynthesis and stomatal conductance on rising forest water-use efficiency. *Proceedings of the National Academy of Sciences, 116*(34), 16909-16914. http://dx.doi.org/10.1073/pnas.1905912116

Hayhoe, K., Wuebbles, D. J., Easterling, D. R., Fahey, D. W., Doherty, S., Kossin, J., et al. (2018). *Our Changing Climate. In Impacts, Risks, and Adaptation in the United States: Fourth National Climate*

Assessment, Volume II Retrieved from Washington, DC, USA:

Humphrey, V., Zscheischler, J., Ciais, P., Gudmundsson, L., Sitch, S., & Seneviratne, S. I. (2018). Sensitivity of atmospheric CO2 growth rate to observed changes in terrestrial water storage. *Nature, 560*(7720), 628-631. http://dx.doi.org/10.1038/s41586-018-0424-4

IPCC. (2018). *Summary for Policymakers. In: Global warming of 1.5°C. An IPCC Special Report on the impacts of global warming of 1.5°C above pre-industrial levels and related global greenhouse gas emission pathways, in the context of strengthening the global response to the threat of climate change, sustainable development, and efforts to eradicate poverty*. Retrieved from Geneva, Switzerland:

Jung, M., Reichstein, M., Schwalm, C. R., Huntingford, C., Sitch, S., Ahlström, A., et al. (2017). Compensatory water effects link yearly global land CO2 sink changes to temperature. *Nature, 541*(7638), 516. http://dx.doi.org/10.1038/nature20780

Lemordant, L., Gentine, P., Stéfanon, M., Drobinski, P., & Fatichi, S. (2016). Modification of land-atmosphere interactions by CO2 effects: Implications for summer dryness and heat wave amplitude. *Geophysical Research Letters, 43*(19). http://dx.doi.org/10.1002/2016GL069896

Lin, Y.-S., Medlyn, B. E., Duursma, R. A., Prentice, C. I., Wang, H., Baig, S., et al. (2015). Optimal stomatal behaviour around the world. *Nature Climate Change, 5*(5), 459-464. http://dx.doi.org/10.1038/nclimate2550

Ove Hoegh-Guldberg, Jacob, D., Taylor, M., Bindi, M., Brown, S., Camilloni, I., et al. (2018). IPCC SR1.5: Chapter 3: Impacts of 1.5ºC global warming on natural and human systems.

Prudhomme, C., Giuntoli, I., Robinson, E. L., Clark, D. B., Arnell, N. W., Dankers, R., et al. (2014). Hydrological droughts in the 21st century, hotspots and uncertainties from a global multimodel ensemble experiment. *Proceedings of the National Academy of Sciences, 111*(9), 3262-3267. http://dx.doi.org/10.1073/pnas.1222473110

Rasmussen, R., Liu, C., Ikeda, K., Gochis, D., Yates, D., Chen, F., et al. (2011). High-Resolution Coupled Climate Runoff Simulations of Seasonal Snowfall over Colorado: A Process Study of Current and Warmer Climate. *24*(12), 3015-3048. https://journals.ametsoc.org/doi/abs/10.1175/2010JCLI3985.1

Reitz, M., & Sanford, W. E. (2019). Estimating quick-flow runoff at the monthly timescale for the conterminous United States. *Journal of Hydrology, 573*, 841-854. http://pubs.er.usgs.gov/publication/70203038

Vose, R. S., Easterling, D. R., Kunkel, K. E., LeGrande, A. N., & Wehner, M. F. (2017). Temperature changes in the United States. In D. J. Wuebbles, D. W. Fahey, K. A. Hibbard, D. J. Dokken, B. C. Stewart, & T. K. Maycock (Eds.), *Climate Science Special Report: Fourth National Climate Assessment, Volume I* (pp. 185-206). Washington, D.C.: U.S. Global Change Research Program.

Xu, C., McDowell, N. G., Sevanto, S., & Phytologist, F.-R. A. (2013). Our limited ability to predict vegetation dynamics under water stress. *New Phytologist*. http://dx.doi.org/10.1111/nph.12450@10.1002/(ISSN)1469-8137(CAT)FeatureIssues(VI)Droughtinducedforestmortality

REVIEWERS' COMMENTS:

Reviewer #2 (Remarks to the Author):

Re-review of manuscript "Dust Tides and Fast Meridional Motions in the Martian Atmosphere During Major Dust Storms" submitted to Nature Communications

I have re-reviewed the revised manuscript. However, I do not think my main point of concern, the transport of water vapor from the polar regions to the low latitudes where it would be available for upward transport in deep dusty convection, has been addressed adequately. While some of the points I had raised concerning this issue have been included into the extended discussion section of the revised manuscript (lines 242-262), the discussion mainly qualifies statements made previously, rather than providing convincing arguments for this process to be relevant. In southern spring water vapor is located at low latitudes no matter whether a dust storm is occurring or not (e.g. supplementary figures 10 a,b) so the process is not really important in this season. In southern summer the water vapor is more concentrated at southern polar latitudes as shown in their supplementary figure 10 c. This pattern is obviously modified by the dust storm in MY 28 (supplementary figure 10 d) but the timing is not favorable. The manuscript states that "the dusty deep convection becomes more widespread during global and regional dust storms at southern midlatitudes" and Heavens et al. (2019) is provided as a reference. While this is not incorrect, it is also true that deep dusty convection increases over a wide latitude range that includes the northern midlatitudes, as evidenced for MY 29 in figure 7 of the aforementioned manuscript and for the MY 34 global dust storm as shown in a recent JGR manuscript by the same author. So I am still not convinced about the relevance of the process for actual water transport in dusty deep convection that is put forward by the manuscript.

The authors have created a new figure that is included in their review response, in which they provide data excerpts from the Mars Climate Database at higher altitudes (20 Pa) as well as for the MY 25 global dust storm. I appreciate the effort. However, while the data for MY 28 shows slightly more water vapor at 20 Pa in the early afternoon than at 50 Pa, it is questionable how systematic this behavior is. The MY 25 data does not show significant increases in water vapor mixing ratio north of 30 S between noon and midnight at either pressure level so I don't see how it helps the argument. The manuscript still does not provide a convincing argument for the claim that these tidal processes are significant for water vapor transport into the middle atmosphere by dusty deep convection. Without this connection solidly established, the work is largely an extension and generalization of the work published on the MY 34 global dust storm, which I would consider interesting for the specialist but not significant for broad audiences. Taking these two findings together I do not consider this manuscript to be suitable for publication in a Nature journal.

Reviewer #3 (Remarks to the Author):

I am satisfied with the current revision.
